# Microbial methionine transporters and biotechnological applications

**DOI:** 10.1007/s00253-021-11307-w

**Published:** 2021-04-30

**Authors:** Nurul Amira Mohammad Mohany, Alessandra Totti, Keith R. Naylor, Harald Janovjak

**Affiliations:** 1 Australian Regenerative Medicine Institute (ARMI), Faculty of Medicine, Nursing and Health Sciences, Monash University, Melbourne, Clayton, Australia; 2 European Molecular Biology Laboratory Australia (EMBL Australia), Monash University, Melbourne, Clayton, Australia; 3 Department of Pharmacy and Biotechnology FaBiT, University of Bologna, Bologna, Italy

**Keywords:** Membrane transport, Amino acids, Solute efflux, Cell engineering, Synthetic biology, Fermentation

## Abstract

**Abstract:**

Methionine (Met) is an essential amino acid with commercial value in animal feed, human nutrition, and as a chemical precursor. Microbial production of Met has seen intensive investigation towards a more sustainable alternative to the chemical synthesis that currently meets the global Met demand. Indeed, efficient Met biosynthesis has been achieved in genetically modified bacteria that harbor engineered enzymes and streamlined metabolic pathways. Very recently, the export of Met as the final step during its fermentative production has been studied and optimized, primarily through identification and expression of microbial Met efflux transporters. In this mini-review, we summarize the current knowledge on four families of Met export and import transporters that have been harnessed for the production of Met and other valuable biomolecules. These families are discussed with respect to their function, gene regulation, and biotechnological applications. We cover methods for identification and characterization of Met transporters as the basis for the further engineering of these proteins and for exploration of other solute carrier families. The available arsenal of Met transporters from different species and protein families provides blueprints not only for fermentative production but also synthetic biology systems, such as molecular sensors and cell-cell communication systems.

**Key points:**

*• Sustainable production of methionine (Met) using microbes is actively explored.*

*• Met transporters of four families increase production yield and specificity.*

*• Further applications include other biosynthetic pathways and synthetic biology.*

## Introduction

**Fig. 1:**
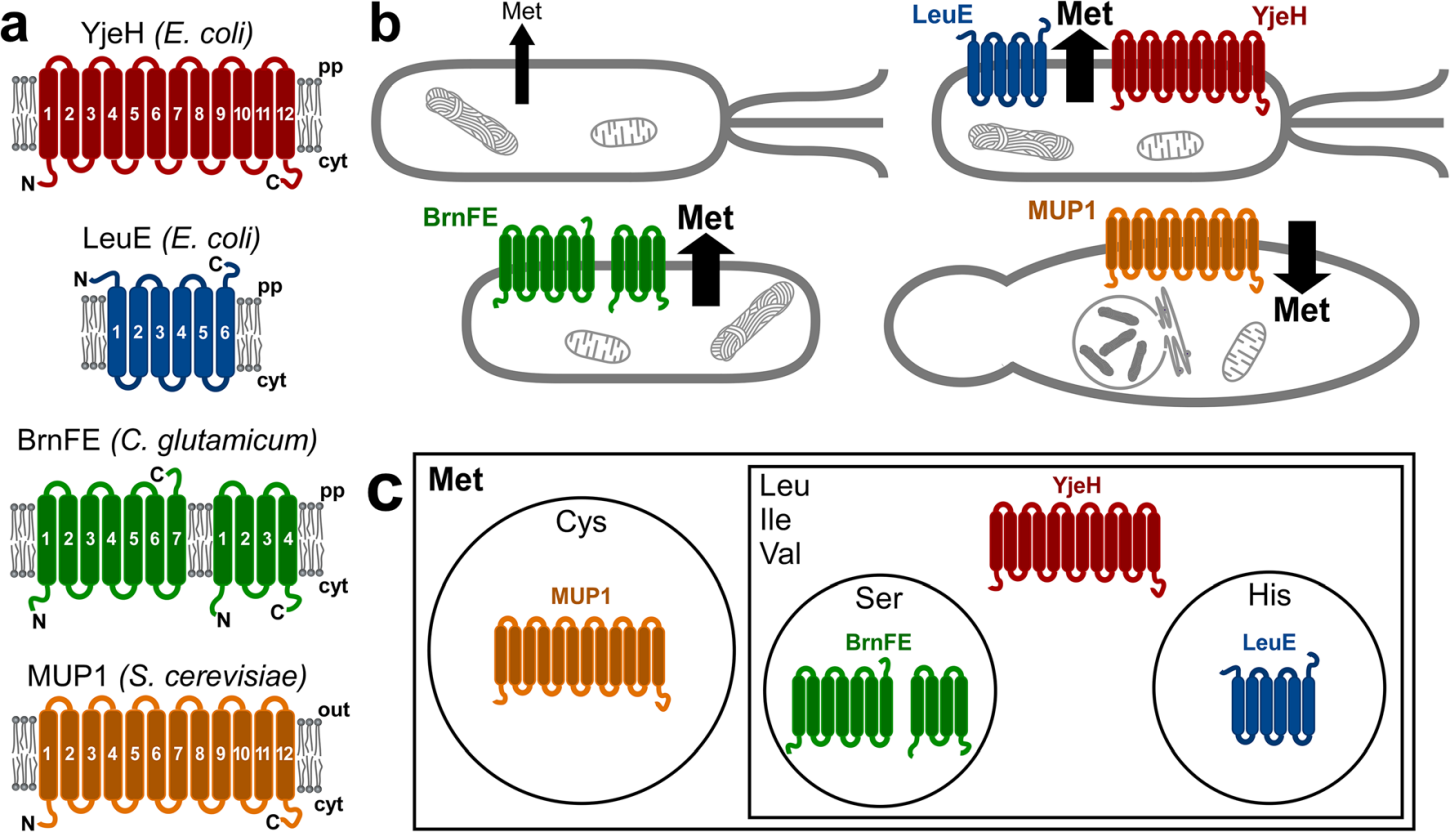
Met transporters harnessed in biomolecule production. **a** Topology and membrane orientation of Met transporters. Pp: periplasm. Cyt: cytosol. Out: Extracellular space. N: N-terminus. C: C-terminus. **b** Transport activities are to increase Met efflux or, in one case, increase Met influx. **c** Known substrates. Transporter cartoons are placed in circles and boxes that represent their substrates. For instance, YjeH is a carrier of Met, Leu, Ile, and Val but not Ser or His

In seminal work dating back ~30 years, Reinhard Krämer and co-workers demonstrated that the excretion of several amino acids, including glutamate, lysine (Lys), and isoleucine (Ile), from *Corynebacterium glutamicum* is an active process (Krämer 1994). These seminal studies initiated a hunt for export carriers for other amino acids in *C. glutamicum* and other bacteria. As early as in 2005 and continuing until more recently, several exporters of the essential amino acid Met have been identified on the gene level, characterized on the protein level and ultimately employed on the level of engineered producer strains (Table 1, Fig. 1). The scope of this review is a comprehensive and timely discussion of the structure, function, and applications of these transporters. We cover three bacterial exporter families which, to the best of our knowledge, entail all studied Met exporters. We also cover a yeast Met importer that has been explored in strain engineering for the production of the Met derivative S-adenosyl-L-methionine (SAM). We are not aware of reviews focusing on microbial Met transporter families, despite recent extensive applications and potential novel developments in synthetic biology. We do not cover bacterial Met uptake systems, such as the *metD* locus ATP binding cassette (ABC) importer MetNIQ (Merlin et al. 2002) or secondary importers (den Hengst et al. 2006), because the manipulation of these importers is not sufficient to increase Met yields (Trotschel et al. 2005; Figge and Dumon-Seignovert 2014). However, alterations of these importers were combined with expression of the transporters reviewed here, and the relevant studies will be discussed. Components of the ABC importer were also employed in molecular sensors, an emerging synthetic biology application that is also covered here.

**Table 1:** Met transporters with biotechnological applications

Name (species)	Family	TC DB identifier	Uniprot identifier(s)	Key reference^a^
YjeH (*E. coli*)	APC	2.A.3.13.1	P39277	Liu et al. (2015)
LeuE (*E. coli*)	RhtB	2.A.76.1.5	P76249	Kutukova et al. (2005)
BrnF/BrnE (*C. glutamicum*)	LIV-E	2.A.78.1.2	H7C682/H7C692	Trotschel et al. (2005)
MUP1 (*S. cerevisiae*)	APC	2.A.3.8.4	P50276	Isnard et al. (1996)

^a^The first research article describing Met transport for this specific protein

Met is one of two sulfur-containing amino acids. As an essential amino acid, Met is required to be supplied in the human diet and linked to metabolism, the immune system, and aging (Martinez et al. 2017). In addition to its function as a building block in proteins and as a precursor of the other sulfur-containing amino acid cysteine, Met derivatives participate in crucial cell biochemical processes. For instance, the Met derivative SAM is the primary biological methyl donor in the synthesis of a variety of biomolecules and a supplement or therapeutic (Schubert et al. 2003; Struck et al. 2012; Mato et al. 2013). Furthermore, Met and SAM are precursors of glutathione, which plays a major role in cellular redox reactions and cellular detoxification (Hayes et al. 2005; Valko et al. 2007). In light of these important biological roles, it is not surprising that Met is commercially valuable, and the current global market size for Met is in excess of 5 billion US$ (Willke 2014; ReportLinker 2020). Major demand for industrially produced Met originates from uses in animal feed, human nutrition, and chemical processing. Met supplementation is required for maximal animal productivity and animal health as it is among the first three limiting amino acids in common poultry, aqua, and swine feed (Jankowski et al. 2014; Nunes et al. 2014). Animal feed supplementation consumes ~95% of the world supply of Met (Willke 2014). The current supply of Met is largely provided by chemical synthesis from methyl mercaptan, acrolein, and hydrogen cyanide in a process that requires significant amounts of organic solvents (Lüssling et al. 1974; Ouchi and Shibuya 1974). Chemical synthesis yields the DL-Met racemate, which is either used directly as feed or converted to the L-isomer for human consumption and further processing. The consumption of fossil resources and generation of hazardous intermediates/waste during conventional Met synthesis, and regulatory limitations of the use of synthetic Met in the production of organic food products, prompted the search for more sustainable processes, including microbial Met production (Kumar and Gomes 2005; Willke 2014).

The production of amino acids by coryneform bacteria as well as by *Escherichia coli* has seen intensive investigation in the past two decades. For Met, strain engineering has been conducted since the early 2000s with the goal of making the process as economical as the chemical synthesis. One classical strain engineering approach is chemical or radiative mutagenesis followed by selection for high amino acid production strains (Nielsen 2001). Indeed, mutagenesis has yielded bacterial strains which are able to produce Lys, Ile, and other amino acids in remarkable amounts (Jetten and Sinskey 1995; Kirchner and Tauch 2003; Wendisch 2020). However, random targeting of genes may have undesired effects on overall physiology and may be limited if multiple genes need to be modified (Park and Lee 2008). In the case of Met, metabolic engineering is a formidable challenge due to a complex biosynthesis with manifold inhibitory feedback loops (Figge 2007; Ferla and Patrick 2014). It was through direct genetic manipulation of enzyme expression and regulation that a several fold increased Met yield has been achieved (Shim et al. 2017; Huang et al. 2018a). Despite patent applications filed by several enterprises, including Ajinomoto Co. Inc. (Japan), BASF SE (Germany), and Evonik Industries AG (Germany) (Willke 2014), to the best our knowledge, CJ CheilJedang Corp. (South Korea) is the first and only commercial provider of Met that is produced on a large scale by bacteria. The corresponding product was recently considered safe in the EU and the USA and approved to market in the EU (Aquilina et al. 2013; FDA 2017; Bampidis et al. 2019; EUCommission 2020).

The intracellular strategies to increase Met-production, such as enzyme and pathway engineering, were recently complemented by expression of membrane exporters. These transporters serve, as detailed below, to promote the export of either the final product or intermediates to optimize overall production flux and gain access to various bioproducts. Transporter function is not only of interest in the context of basic membrane protein function and cell physiology but may also be commercially important because, as described below, significant increases in Met yield have been achieved upon exporter expression in laboratory studies. We here discuss three Met exporters and one Met importer that were all employed in production of Met or its derivative. To identify relevant microbial transporter families, we conducted a literature search in the Web of Science Core Collection using linked substrate terms (“Met” and “methionine”) and transport activity terms (e.g., “transport,” “transporter”) and manually curated 164 hits. We then queried Google Scholar and Google Patents with the identified proteins for further literature/patents and the annotated protein database Uniprot (http://www.uniprot.org; June 2020) to validate that no Met transporter families were missed. For these families, we discuss available knowledge of their function and applications and specify their identifiers in the Transporter Classification (TC) Database (http://www.tcdb.org/). We conclude with a summary of the lessons learned and future perspectives in the orthogonal field of synthetic biology that may harness these carrier proteins.

## YjeH (TC 2.A.3.13.1)

YjeH of *E. coli* is a member of the amino acid efflux (AAE) family (TC 2.A.3.13) within the amino acid-polyamine-organocation (APC) family of transporters (TC 2.A.3). The APC family is the denominating core family within the APC superfamily, which is one of the largest clusters of secondary carrier proteins and includes 18 individual families under its umbrella (Chang et al. 2004; Wong et al. 2012; Vastermark and Saier Jr. 2014; Vastermark et al. 2014). Members of the APC family are found in prokaryotes and eukaryotes and harness electrochemical gradients generated by activate primary pumps.

Already in 2003, the *yjeH* gene was linked to Met export in a patent application of the Wacker Chemicals research consortium (Maier et al. 2003). The *yjeH* gene is a protein of 418 amino acids and, as most members of the APC family, is predicted to contain twelve transmembrane helices with termini located in the cytoplasm. In a recent study (Liu et al. 2015), the three-dimensional structure of YjeH was modeled using the atomic structure of the arginine/agmatine antiporter AdiC as a template. In the context of this model, it needs to be noted that overall sequence identity between YjeH and AdiC is less than 25% and that for some secondary structure elements only weak homology is observed. For instance, in the sequence region spanning the transmembrane helices of AdiC (~20 to 30 amino acids in length), a AdiC:YjeH sequence alignment using the Needleman-Wunsch algorithm or the Muscle algorithm identifies 3 to 9 identical residues. This exemplifies limited sequence similarity and potential accuracy of the model in some parts of the protein, especially if modeling of substrate binding sites is desired. Experimental studies, e.g., employing crystallography or electron microscopy (Drew and Boudker 2016; Seeger 2018; Bosshart and Fotiadis 2019), are required to shed further light on the structure and substrate binding of YjeH and likewise of all other transporters discussed below. Mechanisms of the regulation of YjeH expression are currently poorly understood. Increasing the cytoplasmic concentrations of Met, Ile and leucine (Leu) strongly induced its expression, but repressors or inducers remain unknown (Liu et al. 2015).

YjeH was experimentally determined to be an efficient exporter of Met and of the three branched-chained amino acids (BCAAs) Leu, Ile, and valine (Val) (Liu et al. 2015). Evidence for exporter function was initially obtained in growth assays in the presence of Met analogues. Analogues, such as L-ethionine, are useful tool compounds as their induction of negative feedback loops and/or impediment of protein function results in reduced protein biosynthesis and retardation of culture growth (Rowbury and Woods 1961; Lawrence et al. 1968; Umbarger 1971). Indeed, growth in the presence of the analogues was influenced by *yjeh* deletion (resulting in reduced growth) or (over)expression (resulting in normal or augmented growth) (Liu et al. 2015). The specificity of YjeH was then delineated by providing dipeptides, which enter the cells through unrelated transporters (Payne and Smith 1994) and provide intracellular substrate upon cleavage. When testing the twenty natural amino acids, it was found that only Met, Leu, Ile, and Val demonstrated more efficient export in the reference strain when comparing it to the deletion strain. This export function was inhibited in the presence of carbonyl cyanide m-chlorophenylhydrazone (CCCP), suggesting that YjeH activity depends on a transmembrane proton gradient (Liu et al. 2015).

YjeH has been incorporated in several Met-producing *E. coli* strains. In the study that characterized the protein, a 70% increase in Met yield was achieved (Liu et al. 2015). This result was confirmed in a following study that also employed strains with further modifications, including in *metD*, to increase intracellular Met levels (Huang et al. 2017). A second application of YjeH was to reengineer cellular carbon flux not in the direction of Met but rather O-succinyl-L-serine (OSH) (Huang et al. 2018b; Zhu et al. 2020a). OSH is a biological starting molecule for conversion into commodity chemicals, such as butanols or lactones (Hong et al. 2014). Met export was explored to increase OSH yield because OSH is a precursor of Met and several catalytic steps leading to OSH are repressed by Met. In *E. coli*, these steps include transcriptional regulation by the MetJ repressor as well as feedback inhibition (Ferla and Patrick 2014; Shim et al. 2017). Incorporation of YjeH in a strain that exhibited deletion of *metD* and altered levels of OSH producing enzymes resulted in a 22% increased OSH yield (Huang et al. 2018b; Zhu et al. 2020a). This example demonstrates the ability of a transporter to assist in the reconstruction of cellular metabolic networks for biomolecule production along various nodes of a biosynthetic pathway.

## LeuE (YeaS) (TC 2.A.76.1.5)

LeuE of *E. coli* is a member of the resistance to homoserine (HomoSer)/threonine (RhtB) transporter family (TC 2.A.76) within the Lys exporter (LysE) superfamily (Aleshin et al. 1999; Vrljic et al. 1999). The LysE superfamily is present in Gram-positive bacteria, Gram-negative bacteria, as well as archaea (Tsu and Saier Jr. 2015). The denominating family members catalyze the export of amino acids, e.g., Lys (LysE) and serine/HomoSer (RhtB) (Zakataeva et al. 1999), while other members of the eleven families transport heavy metal ions (Tsu and Saier Jr. 2015).

The *leuE* gene encodes a protein of 212 amino acids. Transmembrane analysis of the primary sequence using TMHMM2.0 (Krogh et al. 2001) reveals that LeuE contains six predicted transmembrane helices. The N- and C-termini are located extracellularly in a topology that is a canonical feature of the LysE family (Tsu and Saier Jr. 2015). LeuE is an efficient Leu transporter with additional substrates that include Met and histidine (His) (Kutukova et al. 2005). The function of LeuE was initially examined by overexpression and gene inactivation in *E. coli*. To identify the amino acid substrate transport profile, resistance of the overexpressor or inactivator strain to amino acids in minimal medium was examined. As already mentioned above, free amino acids and peptides can exhibit toxicity due to inhibition of biosynthetic pathways (Rowbury and Woods 1961; Lawrence et al. 1968; Umbarger 1971). LeuE overexpression conferred strong resistance (>10-fold increase in the minimal inhibitory concentration (MIC)) to three Leu derivatives: the glycyl-l-leucine peptide, L-α-amino-n-butyric acid, and 4-aza-DL-leucine. In addition, milder resistance (5- to 10-fold increase in MIC) was observed to L-norleucine (a Met analogue), DL-methionine sulfone, HomoSer, and His (Eggeling and Sahm 2003; Kutukova et al. 2005). LeuE inactivation in turn resulted in increased sensitivity to a leucine derivative (<0.5-fold decrease in MIC).

To confirm that LeuE acts as an exporter, amino acid accumulation in the growth medium was quantified in overexpressors that were also engineered for increased amino acids synthesis (Kutukova et al. 2005). For all three amino acids tested (Leu, Met, His), increased extracellular levels were observed, with Leu yielding the biggest increase (~4-fold higher levels over a 7-h incubation period) compared to Met and His (~1.5-fold higher levels). It was shown in the same study that addition of CCCP to uncouple the proton gradient across the plasma membrane abolished this gain. Unlike many other RhtB transporters, expression of LeuE was induced by its substrate. The underlying mechanism was release of direct repression by the global Lrp regulator protein (Kutukova et al. 2005; Cho et al. 2008).

Besides in the strain discussed above, LeuE overexpression has not been explored for amino acid production in *E. coli*. However, elevated LeuE expression was observed in *Gluconacetobacter europaeus* strains engineered for amino acid production (Akasaka et al. 2014). *G. europaeus* is an acetic acid-resistant strain employed in the industrial production of vinegars. Several fold increased Leu and Val production was achieved by manipulation of the *G. europaeus* Lrp regulator (Akasaka et al. 2014). This effect was primarily attributed to elevated transcript levels for *G. europaeus* enzymes involved in BCAA carbon flux. Notably, the one Lrp-deleted *G. europaeus* strain that exhibited increased levels of the LeuE homologue suffered from retarded growth, highlighting that excessive amino acid export can be detrimental (as observed for other transporter families; see below).

## BrnFE (TC 2.A.78.1.2)

The BrnF and BrnE transmembrane protein pair, best studied in *C. glutamicum*, is a founding member of the Leu-Ile-Val exporter (LIV-E) family (TC 2.A.78) of BCAA exporters (Kennerknecht et al. 2002; Eggeling and Sahm 2003). *C. glutamicum* is a Gram-positive facultative anaerobic bacterium that is extensively applied in the large-scale production of amino acids (Woo and Park 2014). A notable earlier study (Belitsky et al. 1997) already linked the *B. subtilis* BrnFE orthologues, AzC and AzD, to amino acid transport as their expression conferred resistance to the leucine analogue 4-azaleucine. The LIV-E family is widespread in prokaryotes.

BrnFE was identified by assessing the sensitivity of 1800 *C. glutamicum* transposon mutants to *bis*-Ile-dipeptides (Kennerknecht et al. 2002). The screen yielded three mutants in one of which the transposon was inserted in BrnF. As for other members of the LIV-E family, BrnF is the large component of a protein complex (251 amino acids and seven transmembrane helices) and BrnE the small component (108 amino acids and four transmembrane helices). BrnFE functions as an obligatory protein pair: The overexpression of the individual proteins neither confers resistance to the dipeptides/4-azaleucine nor does it increase amino acid export (Belitsky et al. 1997; Kennerknecht et al. 2002). The addition of valinomycin, which abolishes the electrochemical gradient across a membrane, inhibited efflux, indicating that the BrnFE system is dependent on the proton motive force (Hermann and Kramer 1996). Among the BCAAs, BrnFE transports Leu and Ile more efficiently than Val (Kennerknecht et al. 2002). It was only in later work that BrnFE was also shown to function as a Met and HomoSer exporter (Trotschel et al. 2005; Li et al. 2020). In the case of Met, this function was identified in a complementary approach through microarray analysis of the gene expression profile and functional testing in a Met overproducing *metD*-deficient *C. glutamicum* strain.

The induction of BCAA exporters by their substrates was already appreciated prior to gene identification, e.g., through the demonstration that inhibition of protein synthesis limited induction of export by amino acids (Hermann and Kramer 1996). A Lrp-type transcription factor located adjacent to the BrnFE operon regulates BrnFE expression upon increased intracellular levels of the substrates (Lange et al. 2012). This Lrp-regulator is, unlike the *E. coli* Lrp mentioned above, specific to the BrnFE operon. In an interesting application, the specific nature of this regulation has been harnessed for the development of fluorescent amino acid sensors (Mustafi et al. 2012). It is also noteworthy that BrnFE-deficient *C. glutamicum* mutants no longer excrete BCAAs, whereas they still export Met, suggesting that other transport mechanisms exist for Met (Trotschel et al. 2005).

BrnFE and its *E. coli* orthologues YgaZH have been overexpressed in the production of several amino acids, including Val (Park et al. 2007), Ile (Xie et al. 2012; Yin et al. 2013), HomoSer (Li et al. 2020), and Met (Park et al. 2006; Qin et al. 2015), in either *E. coli* or *C. glutamicum*. In these studies, addition of the exporter to strains with elevated intracellular amino acid levels resulted in increased extracellular amino acid yields by 20 to 50%. However, as pointed out for LeuE before, in some of these studies (Qin et al. 2015: Yin et al., 2013 #999), reduced cell growth upon exporter overexpression was observed. Notable additional information is found in two patents. A patent with a priority date in 2001 (Tabolina et al. 2001) does not examine the function of YgaZH specifically but reports benefits of transporter overexpression in the production of various amino acids, including BCAAs, Met, Thr, and Pro, which suggests a broad amino acid substrate profile for YgaZH. In one later patent by Evonik with a priority date in 2014 (Figge et al. 2014), a systematic approach examined a panel of LIV-E proteins from multiple bacteria for Met production in *E. coli*. Interestingly, overexpression of the *E. coli* protein in *E. coli* yielded no significant increase in Met yield, while overexpression of exporters from other bacteria did achieve this.

## MUP1 (TC 2.A.3.8.4)

Complementary to the bacterial exporters that we discussed above, we also note that a eukaryotic APC-family Met importer has been employed in cell engineering. In *Saccharomyces cerevisiae*, the existence of two uptake systems for Met, one exhibiting high and the other low affinity, was first reported by Gits and Grenson (1967). Two Met transporters were thereafter identified and named MUP1 and MUP3, respectively (Isnard et al. 1996). Along with other amino acid transporters in *S. cerevisiae*, MUP1 and MUP3 belong to the amino APC transporter superfamily (see above) and specifically to the LAT family (TC 2.A.3.8) which is exclusively found in eukaryotes (Jack et al. 2000). MUP1 is the high affinity transporter that has been expressed heterologously for bioproduction and will thus be discussed below. MUP3 (located on chromosome VIII) is the low affinity transporter (apparent Michaelis constant of ~1 mM) that shares with MUP1 a common topology (Isnard et al. 1996) and transcriptional regulation (Menant et al. 2006) but has never been utilized technologically.

The *mup1* gene, located on chromosome VII, encodes a protein of 574 amino acid residues with cytosolic termini and twelve membrane spanning regions (Isnard et al. 1996; Krogh et al. 2001; Henne et al. 2015). The gene was discovered in a study that explored utilization of Met sulfoxide (MetSO) as a sulfur source (Isnard et al. 1996). A mutant strain was identified that was unable to utilize MetSO and also was insensitive to ethionine sulfoxide (EthSO), a toxic Met (SO) analogue. Furthermore, this strain grew poorly on low concentrations of Met and did not exhibit high affinity Met uptake, indicative of the absence of the high affinity transporter. To identify the transporter gene, a yeast genomic library was transformed into this strain followed by growth on MetSO and EthSO containing media. *mup1* identified in this way has been shown to encode for a transporter that is specific for Met (apparent Michaelis constant of ~13 μM) and its analogues (EthSO and MetSO) with no inhibition observed by, e.g., hydrophobic or charged amino acids (Gits and Grenson 1967; Isnard et al. 1996). In addition, MUP1 was assigned to import cysteine (Kosugi et al. 2001). This latter function was demonstrated, similarly to Met import, using a toxic amino acid analogue, uptake measurements, and complementation with a genomic library. Unlike other *S. cerevisiae* transporters capable of Met import, expression of *mup1* is repressed upon exposure to elevated extracellular Met (Menant et al. 2006). In addition, on the protein level, existing MUP1 proteins are rapidly internalized and degraded (Menant et al. 2006; Lin et al. 2008). Both the transcriptional regulation and the protein-level regulation rely on ubiquitination, and analysis of MUP1 internalization has helped elucidate the mechanisms of ubiquitin-mediated protein endocytosis (Lee et al. 2019). In engineered cells, these regulation mechanisms were replaced to enable sustained expression of MUP1.

MUP1 was applied biotechnologically in *Pichia pastoris*. *P. pastoris* is a powerful cell system for protein and metabolite production because it combines elements of eukaryotic cell physiology, such as protein processing and post-translational modifications, with ease of manipulation and growth at high densities in inexpensive media (Pena et al. 2018). *S. cerevisiae* MUP1 was expressed heterologously in *P. pastoris* to improve the yield of SAM production (Ravi Kant et al. 2014). SAM is generated by SAM synthetase from its precursors Met and ATP, and Met supplementation is generally necessary for enhanced SAM biosynthesis (Hu et al. 2007; 2009). MUP1 expression was explored to increase Met import and SAM yield in a strain that already co-overexpressed SAM synthetase 2 and adenylate kinase 1 (ADK1), latter to increase ATP levels (Sakai et al. 1994; 1995). A 33% increase in SAM yield was achieved upon overexpression of MUP1 without a decrease in cell growth (Ravi Kant et al. 2014). Notably, Met availability did not contribute to SAM yield in an intermediary strain that only expressed the synthetase but then was limiting upon increased ATP levels. This example highlights the intricate interplay of multiple components within biosynthetic pathways. It will be interesting to explore to what extent engineering of intracellular Met fluxes (as demonstrated for bacteria above) and increased import of MUP1 may act synergistically on SAM production.

## Conclusions

Commercially valuable biomolecules, including amino acids (Jetten and Sinskey 1995; Kirchner and Tauch 2003; Wendisch 2020), have been produced using microbes for several decades. Microbial Met production, which is subject to complex biosynthesis including manifold feedback loops, has been approached by extensive enzyme and metabolic engineering as well as optimization of fermentation conditions (Willke 2014; Shim et al. 2017; Huang et al. 2018a). This approach offers “green” access to Met, and a first commercial Met feed additive generated using genetically modified bacteria has reached market approval (see above). Complementary to the intracellular flux optimization, it was realized that transport of Met across the membrane may be relevant to optimize production, alike to observations in other bioproducts (Kell et al. 2015; Zhu et al. 2020b). Starting from seminal studies of microbial amino acid export in the 1990s, Met exporter genes were identified, characterized, and applied in engineered strains since. Results from the laboratory studies reviewed above indicate that a significant increase in Met yield can be achieved upon transporter overexpression in amino acid producer strains. In addition to their biotechnological use, amino acid exporters are also of fundamental biology interest because of the important roles they play in bacterial physiology, such as to balance intracellular amino acid pools.

We here reviewed the three known Met exporter families that have been explored—with varying degrees of intensity—to increase Met yields. Albeit these proteins belong to three disparate protein families they have in common that they also export other amino acids, in particular BCAAs. In at least one protein family (BrnFE), this promiscuity has been harnessed with this gene incorporated in producer strains for multiple amino acids (see above) (Tabolina et al. 2001). In light of future developments, it is noteworthy that different approaches were applied to identify these transporters. In the case of *yjeH* and *leuE*, pre-existing knowledge that these genes encode members of a transporter family prompted them to be studied with a panel of amino acids. In the case of *brnFE* and *mup1*, genes were identified by knock-out using transposon insertion or random mutagenesis followed by growth on a selection medium and/or complementation. These studies showcase an arsenal of molecular methods for transporter identification and characterization that may collectively serve as a template for studies into carriers of other solutes. One further noteworthy recent development was that a Met exporter (YjeH) was not only applied for Met production but to release intracellular inhibition of enzymes in the synthesis of a different metabolite (Huang et al. 2018b; Zhu et al. 2020a). Along with the example of increased Met import by MUP1 to provide a reaction precursor (Ravi Kant et al. 2014), this work demonstrates the versatility of transporters in the reconstruction of cellular metabolic fluxes to engineer even better producer strains.

## Future perspectives

Strategies that have proven effective in the optimization of other transporters, such as fine-tuning of expression levels or improved transporter function (Kell et al. 2015; Zhu et al. 2020b), have generally not yet been applied to Met exporters. It needs to be considered that high expression levels of these proteins can result in an excess substrate release that may negatively affect viability. Furthermore, limited structural information and challenges in homology modeling may hamper molecular reengineering, e.g., towards varying affinities or substrate specificities, of these and many other transporter families (Bill et al. 2011; Drew and Boudker 2016; Cheng 2018; Seeger 2018; Bosshart and Fotiadis 2019). While there is evidence from other membrane protein classes that complex gating processes can be reengineered without structural information (e.g., in ion channels and receptors (Armbruster et al. 2007; Janovjak et al. 2010)), rational design generally requires knowledge with atomic resolution. In the absence of rational protein design, transporter mining from published genomes and directed evolution may prove a valuable alternative to identify novel and useful functions (Kell et al. 2015; Zhu et al. 2020b). Functional information of Met exporters are, with very few exceptions (Figge et al. 2014), only available for *E. coli* and *C. glutamicum*. This may not be surprising given that these two organisms are the major amino acid producers. However, it is evident from available numerous genome sequences that exporter are widespread motivating systematic exploration. In one work, such exploration achieved higher production levels upon heterologous expression of orthologues from other bacterial species (Figge et al. 2014). In general, successes achieved in the engineering of transporters for other microbial production processes (Kell et al. 2015; Zhu et al. 2020b) may guide further improvement of Met transporters.

Engineered Met transporters and their domains also find applications outside of Met production, such as in synthetic biology. For instance, whilst it was shown that manipulation of the MetNIQ ABC importer alone is not sufficient to alter Met production (Trotschel et al. 2005; Figge and Dumon-Seignovert 2014), and its Met binding domains have been used as templates to engineer Met biosensors in two studies (Mohsin and Ahmad 2014; Ko and Lee 2019). The MetN subunit was genetically fused to a copy of the cyan fluorescent protein and the yellow fluorescent protein (YFP) to form the Förster resonance energy transfer (FRET)-based sensor FLIPM (Mohsin and Ahmad 2014). FLIPM specifically responds to Met binding with changes in the FRET ratio (up to 30%) and has been visualized in real time and even in single cells (Mohsin and Ahmad 2014). A second, more recent study demonstrated the use of the MetQ subunit in a semi-synthetic FRET sensor (Ko and Lee 2019). A UV-sensitive coumarin-type fluorophore was introduced at four different sites of a MetQ-YFP fusion protein as an unnatural amino acid. All four variants showed increased FRET ratios with the most potent variant displaying an impressive 2.7-fold change. This fusion protein was also responsive to D-Met and glutamine albeit at considerably higher concentrations than for Met, and higher selectivity for Met was achieved through a rational mutagenesis strategy (Ko and Lee 2019). In the future, these biosensors may be further improved, e.g., by incorporating fluorophores that are excited by visible rather than potentially harmful UV light (Suzuki et al. 2012; Mitchell et al. 2017), and ultimately applied to better understand intracellular Met fluxes (Mustafi et al. 2012; Kell et al. 2015).

A further potential synthetic biology application of Met transporters is in synthetic cell-cell communication systems. A role of RhtB/LysE family transporters in bacterial cell-cell communication was proposed (Zakataeva et al. 2006), and amino acids have already been re-purposed as synthetic signaling molecules in mammalian cells (Weber et al. 2009; Bacchus et al. 2012; Williams et al. 2013). The inclusion of membrane transporters in synthetic systems may offer new experimental opportunities. For instance, amino acid signaling molecules generally rely on uptake into cells and this process could be “gated” through the targeted expression of a suited transporter. In a different example, application of microbial transporters in the animal nervous system has been proposed and pursued by us. Information processing in the nervous system occurs in concerted synaptic neurotransmission events that rely on endogenous transporters (e.g., for the re-uptake of neurotransmitters into synaptic vesicles). Microbial transporters could be explored to modulate these processes. For instance, targeting microbial Met exporters to synaptic vesicles may allow dampening the natural neurotransmitter re-uptake or even fully replace vesicle content. The emerging method for manipulation of synaptic vesicle function promises new insights into nervous system information processing (Karpova et al. 2005; Lin et al. 2013; Rost et al. 2015). Some open questions remain towards this method: Is expression of a microbial membrane protein in an animal neuron possible? Notably, neuronal delivery of microbial ion channels and pumps (Rost et al. 2017), or even their subdomains (Janovjak et al. 2010), is routinely performed providing a robust methodological basis. Furthermore, targeting of microbial pumps to vesicles has been achieved (Rost et al. 2015) and will be further aided by new methods to study vesicles (McKenzie et al. 2019). And why would Met and Met transporters be the choice for such endeavor? Firstly, as described above, Met transport function has been established in multiple carrier protein families, and the ability to explore proteins of different origins can increase success chances in synthetic biology (Chow et al. 2010). Secondly, Met itself is a well-suited ligand because it is tolerated at high doses by animal tissues (Stefanello et al. 2007; de Rezende and D'Almeida 2014). These examples highlight how a portfolio of characterized transmembrane carriers may lead to new developments and discoveries across fields and disciplines.
